# Role of Human Metapneumovirus, Influenza A Virus and Respiratory Syncytial Virus in Causing WHO-Defined Severe Pneumonia in Children in a Developing Country

**DOI:** 10.1371/journal.pone.0074756

**Published:** 2013-09-18

**Authors:** Asad Ali, Asif Raza Khowaja, Maaman Zahoor Bashir, Fatima Aziz, Sultan Mustafa, Anita Zaidi

**Affiliations:** 1 Department of Paediatrics and Child Health, Aga Khan University, Karachi, Pakistan; 2 Abbasi Shaheed Hospital, Karachi, Pakistan; University of Liverpool, United Kingdom

## Abstract

**Objective:**

The role of respiratory viruses in causing severe, life threatening pneumonia in children in developing countries is not well established. Our study aims to determine the role of human metapneumovirus (HMPV), influenza A virus and respiratory syncytial virus (RSV) in children, aged 6 weeks to 2 years, hospitalized with WHO defined severe pneumonia (tachypnea plus any general danger sign or chest in-drawing) at a public sector hospital in Karachi, Pakistan.

**Methods:**

This study was conducted from November 2010 to September 2011 at Abbassi Shaheed Hospital, a large public tertiary care hospital in Karachi, Pakistan. Children admitted with WHO-defined severe pneumonia were enrolled and throat swabs were obtained to detect respiratory viruses using real time RT-PCR. Chest x-rays of all subjects were obtained and independently interpreted by two radiologists to diagnose radiologic pneumonia.

**Results:**

169 children were enrolled. HMPV was detected in 24 (14.2%), influenza A virus in 9 (5.3%) and RSV in 30 (17.8%) children admitted with severe pneumonia. Of 9 patients with influenza A, 8 tested positive for H1N1. Viral etiology was found in 18% of radiologically confirmed pneumonia. HMPV infections peaked in February and April, influenza A was prevalent in January, June and November and RSV infections were most prevalent from June to September.

**Conclusion:**

HMPV, influenza A and RSV are common causes of WHO-defined severe pneumonia in hospitalized children in Karachi. Knowledge regarding the viral etiology of pediatric pneumonia and individual viral seasonality can help in the recommendation and implementation of appropriate management strategies.

## Introduction

Pneumonia is the second leading cause of childhood mortality worldwide, with estimated 1.25 million deaths per year [1]. In Pakistan, the estimated community based incidence of pneumonia in children aged less than five years is 1–4 episodes per 100 children per year, which accounts for 13% of all under five deaths every year [2]. While the role of respiratory viruses including HMPV, influenza A and RSV is well established in causing self-limiting upper respiratory tract infections or mild pneumonia, their contribution in causing severe and radiologically proven pneumonia in developing countries is less clear [3]. This knowledge is critical to guide the development of preventive and therapeutic interventions against these viruses, and to make a case for the use of existing interventions like the influenza vaccine [4].

A challenge in finding the viral etiology of severe pneumonia in developing countries is the general unavailability of appropriate diagnostic facilities [5]. Even if diagnostic tests are available, they are restricted to high resource, private tertiary care hospitals, whereas most of the deaths due to pneumonia occur in the community or in public sector facilities [2]. To determine the etiology of severe, potentially fatal pneumonia in developing countries, studies need to be conducted in health care facilities which cater to the lower income population, which is at the highest risk for adverse health outcomes. We therefore conducted a longitudinal surveillance study at a busy public sector hospital in Karachi to determine the role of respiratory viruses in WHO-defined severe pneumonia, including radiologically proven pneumonia, in children 6 weeks to 2 years of age.

## Methods

This was an 11 month long study conducted between November 2010 and September 2011 at Abbasi Shaheed Hospital in Karachi, Pakistan. Abbassi Shaheed is a public sector hospital that serves the residents of the northern part of Karachi with an estimated population of nearly 1 million. Karachi is located on the coast of Arabian sea and has a relatively mild climate. The latitude and longitude of Karachi are 24.8508° N, 67.0181°E.The city has two main seasons; summer and winter, and receives the monsoon rains from July to September. The humidity levels usually remain high from March to November, while very low in winter. This study covers both seasonal periods.

In this study, WHO defined criteria for severe pneumonia, i.e tachypnea (respiratory rate >60/min in children <2 months, 50/min in children 2–12 months and >40/min in children >12 months) and chest indrawing or any other danger sign [6], were used to identify children 6 weeks to 2 years old, hospitalized at the Abbasi Shaheed Hospital. Children were excluded if they were previously enrolled in the study or if their parents refused to participate. This study received ethical approval from Ethics Review Committee of Aga Khan University, Karachi Pakistan. After written informed consent from the parents or guardians, a baseline questionnaire was completed and pharyngeal swabs were obtained by the study physician. These were transported to the Infectious Diseases Research lab (IDRL) at the Aga Khan University (AKU) for viral testing using real time RT-PCR. The proportion of severe pneumonia cases associated with HMPV, influenza A and RSV was determined. In addition, chest x-rays of all enrolled subjects were obtained and emailed to two radiologists at AKU for interpretation using WHO standard definition for substantial alveolar consolidation i.e. radiologically confirmed pneumonia [7]. These two radiologists were not given any clinical information about the child, and were blinded to each other’s interpretations. A third radiologist was used in case of discordant interpretation of the first two radiologists [7]. Descriptive analysis was done to calculate median age of children, and proportion of respiratory virus etiology. Associations between clinical manifestations and etiological agents were analyzed using chi square test for categorical data and t-test for continuous data, considering a P-value <0.05 as significant value. Statistical analysis was calculated using Stata software (Version 11).

### PCR Analysis

A throat swab was obtained using a commercial flocked swab (Diagnostics Hybrid Inc.) in Universal Transport Medium (Diagnostics Hybrid Inc.) and was transported to the IDRL. RNA was extracted from frozen aliquots (stored at −80C) using QIAamp Viral RNA Mini Kit (Qiagen).Later, real time RT-PCR assays were performed using primers and probes for HMPV [CDC Reference Number: I-036-06], RSV [8], influenza A and A(H1N1)pdm09 [9]. All samples were first tested for influenza A, and those testing positive for influenza A were subsequently tested for A(H1N1)pdm09. Influenza B was not tested. Positive and negative controls were used in each run, and influenza A positive samples were re-confirmed and serotyped at a reference laboratory at North American Medical Research Unit 3 in Cairo, Egypt or in the reference lab of National Institute of Health, Pakistan.

## Results

From November 2010 to September 2011, 387 children between the age of 6 weeks and 2 years with signs suggestive of severe pneumonia were screened. 247(64%) of these were found to be eligible for the study, out of which 169(68%) children with WHO-defined severe pneumonia were enrolled. The demographic characteristics of children who tested positive for viruses are summarized in table 1. The median age of the study subjects was 6 months (IQR 3–9 months). At least one of the three viruses was detected in 61 (36%) of the enrolled subjects. HMPV was detected in 24 (14.2%), influenza A virus in 9 (5.3%) and RSV in 30 (17.8%) subjects. Out of the 9 patients with influenza A, 8 tested positive for H1N1. The clinical features and risk factors among children with each viral etiology is summarized in table 2. Inability to drink was a prominent feature in HMPV (62.5%) and influenza A (44.4%) infected patients, whereas wheezing (64.3%) was most noticeable in RSV infected individuals. Wheezing was found less frequently in patients with influenza A (33.3%) and HMPV (33.3%). Antibiotics were used prior to admission in 69.6% of HMPV, 55.6% of influenza A and 86.7% of RSV positive patients. Majority of children infected with influenza A were found to have smokers in the house (55.6%, p = 0.042).

**Table 1:** A comparison of baseline characteristics of HMPV, Influenza A and RSV infected children.

Baseline characteristics	HMPV* (n = 24)	Influenza A (n = 9)	RSV** (n = 30)
**Age in months (median, IQR)**	6(4–10)	10(6–11)	5(3–7)
**Male**	13 (54.2%)	3 (33.3%)	20 (69%)
**Smokers in the house**	3 (12.5%)	5 (55.6%)	5 (16.7%)
**Number of household members (median, IQR)**	7.5(7–11)	7 (6–8)	6(5–11)
**Ever Vaccinated**	21 (87.5%)	6(66.67%)	19(63.3%)

Abbreviations:

* HMPV: Human metapneumovirus.

** RSV: Respiratory syncitial virus.

**Table 2:** A comparison of the clinical feat ures of HMPV, Influenza A and RSV infected children.

Clinical features	HMPV*		p-value	Influenza A		p-value	RSV**		p-value
	Positive (n = 24)	Negative (n = 145)		Positive (n = 9)	Negative (n = 160)		Positive (n = 30)	Negative (n = 139)	
**Wheezing/noisy breathing**	8(33.3%)	93(64.1%)	0.003	3(33.3%)	98(61.2%)	0.082	18(64.3%)	83(59.7%)	0.682
**Chest indrawing**	16(66.7%)	60(41.4%)	0.021	6(66.7%)	70(43.8%)	0.179	12(40%)	64(46%)	0.546
**Unable to drink**	15(62.5%)	49(33.8%)	0.007	4(44.4%)	60(37.5%)	0.676	10(33.3%)	54(38.9%)	0.572
**Difficult to arouse/abnormally** **sleepy**	5(20.8%)	45(31%)	0.310	3(33.3%)	47(29.4%)	0.800	6(20%)	44(31.6%)	0.205
**Radiologically confirmed** **pneumonia**	2(8.3%)	49(33.7%)	0.01	1(11.11%)	50(31.2%)	0.190	6(20.7%)	45(32.3%)	0.197

Abbreviations:

* HMPV: Human metapneumovirus.

** RSV: Respiratory syncitial virus.

Pneumonia was radiologically confirmed in 51 (30.1%) of the study subjects. Of these, 9 (18%) were positive for at least one of the three viruses tested. HMPV was detected in 2 (4%), influenza A in 1 (2%) and RSV in 6 (12%) of the radiologically confirmed pneumonia cases. Radiologically confirmed pneumonia was found in 2/24 (8.3%) HMPV, 1/9 (11%) influenza A and 6/30 (20%) RSV infected children.

**Figure 1:**
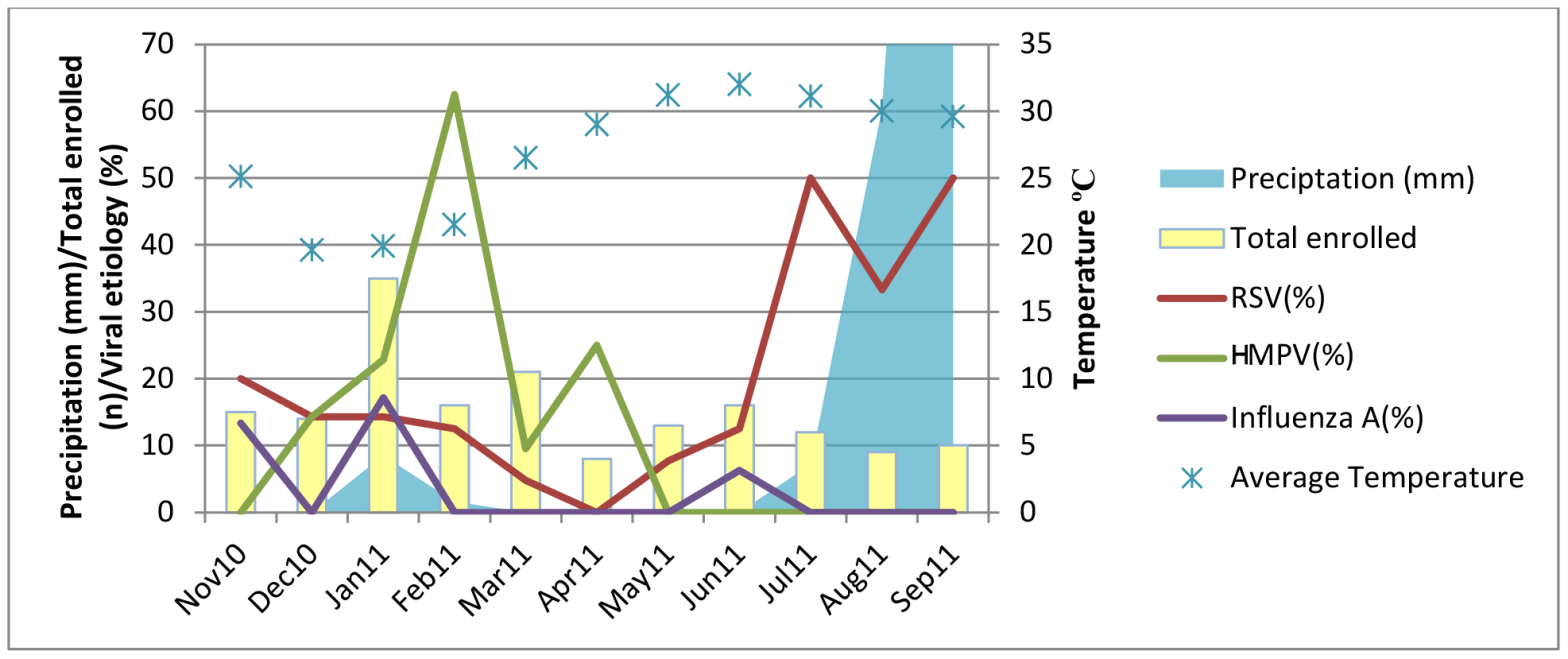
Seasonal distribution of RSV, HMPV and Influenza A cases from Nov 2010 to Sep 2011 at Abbassi Shaheed Hospital, Karachi, Pakistan.

The monthly distribution of each of the three viruses and the corresponding temperature and precipitation data are shown in figure 1. The highest number of rainy days was observed in the months of July, August and September. The lowest temperatures were seen from December to January and the highest from May to July. Most infections with RSV were reported from June to September 2011. The peak incidence of RSV was in July (n = 6, 50%) and September (n = 5, 50%). HMPV showed one major peak in February (n = 10, 63%) and one minor peak in April (n = 2, 25%), and was absent from May to November. Influenza A showed three minor peaks, in January (n = 6, 17%), June (n = 1, 6%) and November (n = 2, 13%) and was not detected during the rest of the year.

## Discussion

This study is the first to describe the viral etiology of WHO defined severe pneumonia in hospitalized children in Pakistan using real time RT-PCR assays. We found that 36% of patients with severe pneumonia were positive for HMPV, influenza A or RSV. This finding is similar to other hospital-based etiological studies done in Bangladesh, India, Gambia and central Australia [10], [11]. On average, viruses are generally isolated in approximately 30–50% of the cases of severe pneumonia hospitalized in developing countries [12].

RSV was the predominant virus in our study. It was isolated from 30 (17%) children, reinforcing the results of several studies in other countries which have demonstrated a similar magnitude of prevalence of this virus in childhood pneumonia [13], [14]. In a study conducted in 2006 in rural Bangladesh on children aged 0–24 months, RSV was found in 21 out of 58(36%) cases admitted with pneumonia [15]. The Bangladesh study however, included cases of non-severe pneumonia, which may account for the difference. A study conducted in rural Kenya identified 16.5% cases of RSV associated severe pneumonia, which is consistent with our findings [14].

We found that 20% of RSV positive, severe pneumonia cases in our study had radiologically confirmed pneumonia. This is lower than what was found in a similar study in Bangladesh (66.7%) [11], [14]. Our strict radiological interpretation was perhaps a factor in the lower proportion of radiologically confirmed pneumonia seen in our study. Nonetheless, RSV positive cases had the highest proportion of radiologically confirmed pneumonia (20%) as compared to other viral causes in our study i.e HMPV (8%) and influenza A(11%).While the possibility of secondary bacterial infection cannot be ruled out, our study shows that viruses are often associated with radiologically confirmed pneumonia.

RSV epidemics generally occur during the winter and spring months, though there is considerable variation [4]. A study conducted in a refugee population in Thailand showed a highly seasonal pattern of RSV infections, occurring predominantly in October each year, just after the peak rainfall [16]. We found RSV infections year round in our study with peaks from July to September, perhaps due to the heavy rainfall observed during this period. As shown in Fig. 1, the highest precipitation occurred in the months of July, August and September, which corresponds with the RSV peak incidence. This is an important association and makes this season an important target for intervention. RSV vaccination or prophylaxis, once available, may offer considerable public health benefit for vulnerable population in this season.

HMPV was found in 14% of children hospitalized with severe pneumonia in our study. This is a higher burden compared to what was recently shown in a prospective 5 year surveillance study in the United States, where only 6% of the hospitalized children tested positive for HMPV [17]. A similar study conducted in Israel found HMPV in 13% of the children hospitalized with pneumonia [18]. Though HMPV seasonality is not well-defined, some studies point towards it being more prominent in the winter season. Majority of cases in the prospective surveillance conducted in the United States were found during the months of January through April [17]. A study in Italy showed that a high incidence of HMPV infection (25.3%) was observed during the 2005–2006 winter-spring season, whereas a much lower rate of infection (4.7%) was found during the next season [19]. As observed in figure 1, the lowest temperatures in Karachi were seen from the months of December to February, which parallel a relatively higher incidence of HMPV infections detected in our study.

The incidence of influenza-virus-associated severe pneumonia requiring hospitalization is important because influenza is an infection that can be prevented with vaccines [20]. Infection with influenza virus predisposes children to infections with other common organisms associated with severe illness, such as pneumococcal and staphylococcal pneumonia [21]. During the year our study was conducted, influenza A was found at a lower rate as compared to RSV and HMPV. The incidence of influenza A in our study (5%) was similar to many other studies conducted in various parts of the world. In a study conducted in El Salvador from 2008–2010, 608 cases of severe pneumonia were tested for respiratory viruses and 37 (6%) were positive for influenza virus [22]. A slight peak in the incidence of influenza in January and February in our study coincides with a lower temperature observed during these months, as seen in figure 1.

Our study has several limitations. This surveillance was conducted for 11 months and the seasonal pattern of respiratory viruses may vary from year to year. Multiple year surveillance is needed to establish seasonality of different respiratory viruses reliably. However, the seasonal pattern seen in our study is consistent with data from other regional countries. While testing for influenza, we only tested for influenza A and not for influenza B. We did not try to elicit bacterial etiology of severe pneumonia in our patients, though all our study subjects received antibiotics. Hence estimates of viral-bacterial co-infections cannot be made. Nonetheless, our study shows that either alone or as part of viral-bacterial co-infections, respiratory viruses play an important role in clinically severe pneumonia in children in Pakistan.

In summary, we found that a considerable proportion of WHO defined severe pneumonia and radiologically proven pneumonia hospitalizations in children in Pakistan are associated with respiratory viruses. Respiratory viruses should be the focus of additional efforts to decrease pneumonia-associated morbidity and mortality in developing countries and defining the burden of these viruses using reliable and sensitive diagnostic tests is an important first step in this regard. Many children with typical respiratory viral symptoms are not brought to the hospital until a secondary bacterial infection develops. A preventive vaccine given at the correct time could prevent many secondary bacterial infections. While our study shows important trends, subsequent year surveillance is also needed to assess if this seasonal pattern is consistent in this region.

## References

[1] (2012) Levels and Trends in Child Mortality. UNICEF, WHO, The World Bank, UN DESA/Population Division.

[2] (2008) Pakistan Demographic and Health Survey 2006–07. Islamabad, Pakistan: National Institute of Population Studies and Macro International Inc.

[3] Ruuskanen O, Lahti E, Jennings LC, Murdoch DR (2011) Viral pneumonia. Lancet 377: 1264–1275. 21435708 10.1016/S0140-6736(10)61459-6 PMC7138033

[4] Weber MW, Mulholland EK, Greenwood BM (1998) Respiratory syncytial virus infection in tropical and developing countries. Trop Med Int Health 3: 268–280. 9623927 10.1046/j.1365-3156.1998.00213.x

[5] Pang T, Peeling RW (2007) Diagnostic tests for infectious diseases in the developing world: two sides of the coin. Trans R Soc Trop Med Hyg 101: 856–857. 17544047 10.1016/j.trstmh.2007.04.014

[6] (2005) Hand Book Integrated Management of Childhood Illnesses: World Health Organization.

[7] (2001) Standardization of Interpretation of Chest Radiographs for the Diagnosis of Pneumonia in Children. World Health Organization. Pneumonia Vaccine Trial Investigators’ Group.

[8] Mentel R, Wegner U, Bruns R, Gurtler L (2003) Real-time PCR to improve the diagnosis of respiratory syncytial virus infection. J Med Microbiol 52: 893–896. 12972584 10.1099/jmm.0.05290-0

[9] Selvaraju SB, Selvarangan R (2010) Evaluation of three influenza A and B real-time reverse transcription-PCR assays and a new 2009 H1N1 assay for detection of influenza viruses. J Clin Microbiol 48: 3870–3875. 20844230 10.1128/JCM.02464-09 PMC3020824

[10] Torzillo P, Dixon J, Manning K, Hutton S, Gratten M, et al (1999) Etiology of acute lower respiratory tract infection in Central Australian Aboriginal children. Pediatr Infect Dis J 18: 714–721. 10462342 10.1097/00006454-199908000-00012

[11] Hasan K, Jolly P, Marquis G, Roy E, Podder G, et al (2006) Viral etiology of pneumonia in a cohort of newborns till 24 months of age in Rural Mirzapur, Bangladesh. Scand J Infect Dis 38: 690–695. 16857616 10.1080/00365540600606473

[12] Forgie IM, O’Neill KP, Lloyd-Evans N, Leinonen M, Campbell H, et al (1991) Etiology of acute lower respiratory tract infections in Gambian children: II. Acute lower respiratory tract infection in children ages one to nine years presenting at the hospital. Pediatr Infect Dis J 10: 42–47. 2003054 10.1097/00006454-199101000-00009

[13] Nokes D (2007) Respiratory Syncytial Virus disease burden in the developing world. In: PA C, editor. Perspectives in Medical Virology. Amsterdam: Elsevier. 183–230.

[14] Nokes DJ, Ngama M, Bett A, Abwao J, Munywoki P, et al (2009) Incidence and severity of respiratory syncytial virus pneumonia in rural Kenyan children identified through hospital surveillance. Clin Infect Dis 49: 1341–1349. 19788358 10.1086/606055 PMC2762474

[15] John TJ, Cherian T, Steinhoff MC, Simoes EA, John M (1991) Etiology of acute respiratory infections in children in tropical southern India. Rev Infect Dis 13 Suppl 6 S463–469. 1862277 10.1093/clinids/13.supplement_6.s463

[16] Turner C, Turner P, Cararra V, Eh Lwe N, Watthanaworawit W, et al (2012) A high burden of respiratory syncytial virus associated pneumonia in children less than two years of age in a South East Asian refugee population. PLoS One 7: e50100. 23185545 10.1371/journal.pone.0050100 PMC3502361

[17] Edwards KM, Zhu Y, Griffin MR, Weinberg GA, Hall CB, et al (2013) Burden of human metapneumovirus infection in young children. N Engl J Med 368: 633–643. 23406028 10.1056/NEJMoa1204630 PMC3662802

[18] Wolf DG, Greenberg D, Kalkstein D, Shemer-Avni Y, Givon-Lavi N, et al (2006) Comparison of human metapneumovirus, respiratory syncytial virus and influenza A virus lower respiratory tract infections in hospitalized young children. Pediatr Infect Dis J 25: 320–324. 16567983 10.1097/01.inf.0000207395.80657.cf

[19] Caracciolo S, Minini C, Colombrita D, Rossi D, Miglietti N, et al (2008) Human metapneumovirus infection in young children hospitalized with acute respiratory tract disease: virologic and clinical features. Pediatr Infect Dis J 27: 406–412. 18382388 10.1097/INF.0b013e318162a164

[20] (2008) The Global Burden of Disease-2004 Update. Geneva: World Health Organization.

[21] Brooks WA, Goswami D, Rahman M, Nahar K, Fry AM, et al (2010) Influenza is a major contributor to childhood pneumonia in a tropical developing country. Pediatr Infect Dis J 29: 216–221. 20190613 10.1097/INF.0b013e3181bc23fd

[22] Clara W, Armero J, Rodriguez D, de Lozano C, Bonilla L, et al (2012) Estimated incidence of influenza-virus-associated severe pneumonia in children in El Salvador, 2008–2010. Bull World Health Organ 90: 756–763. 23109743 10.2471/BLT.11.098202 PMC3471049

